# Enhancing a High-reliability Safety Culture through a Reimagined Leadership Workshop Series

**DOI:** 10.1097/pq9.0000000000000766

**Published:** 2024-09-25

**Authors:** Allison Starr, Shi Yuan Susan Hu, Maitreya Coffey, Daniela D’Annunzio, David During, Bonnie Fleming-Carroll, Richard Wray

**Affiliations:** From the Hospital for Sick Children (SickKids).

## Background:

**Fig. 1.**
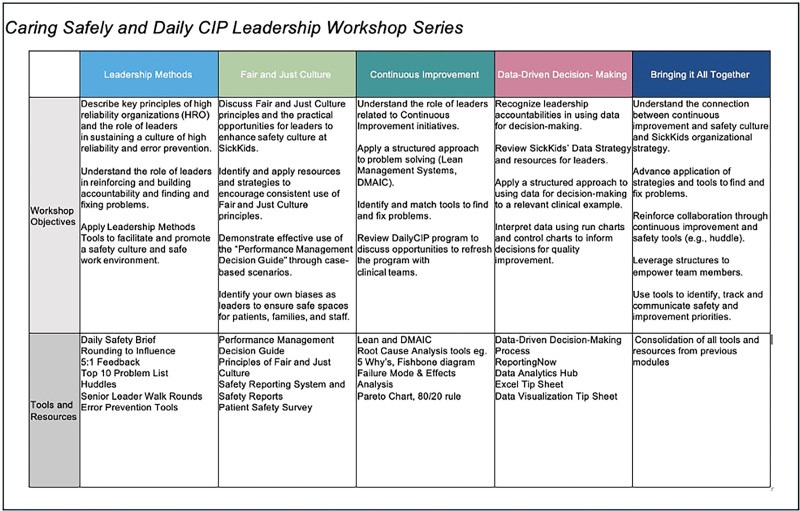
Workshop objectives and outcomes.

Two distinct safety and quality improvement programs are in place for leaders at the Hospital for Sick Children (SickKids). The Daily Continuous Improvement Program, inspired by Thedacare’s lean management system, was implemented in 2012. Additionally, in 2014, SickKids launched a safety-focused initiative called Caring Safely by joining the Solutions for Patient Safety Network. To bolster the high reliability safety culture at SickKids since the launch of these two programs, a needs assessment, guided by the Institute for Healthcare Improvement Framework for Safe, Reliable, and Effective Care, identified an opportunity to integrate content from both programs to revitalize training materials and align theoretical approaches and tools for leadership teams (Fig. 1). The goal was to deliver an educational workshop series to cohesive leadership teams within a 6-month period to enhance uptake of the content and encourage a collaborative approach to applying tools into practice. The desired outcome was to streamline the core concepts from the legacy programs, addressing knowledge gaps and supporting leaders in effectively implementing these principles to enhance the safety culture at SickKids.

## Methods:

**Fig. 2.**
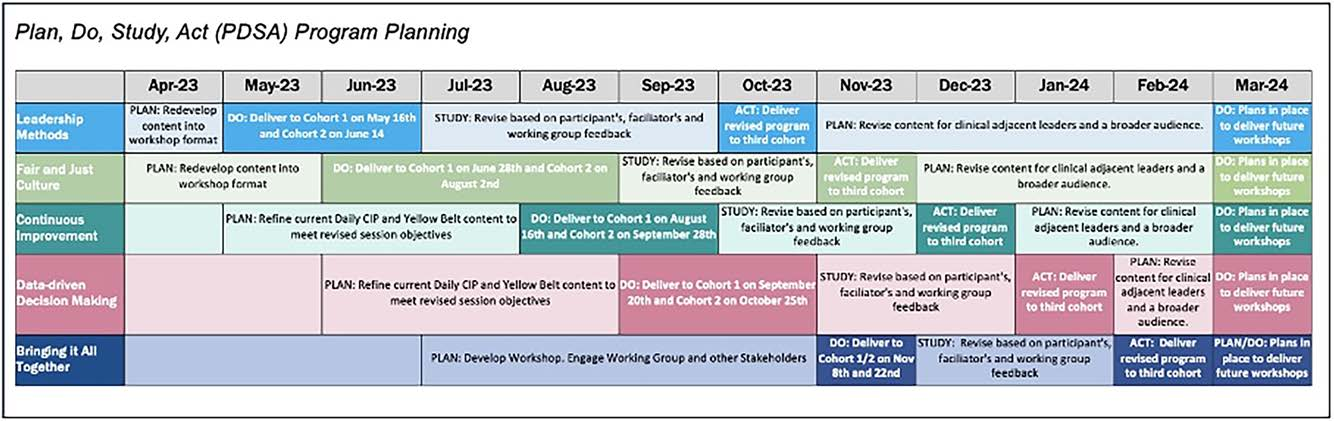
The PDSA approach to workshop planning and refining content for each cohort.

Participants were divided into three cohorts to attend the five-module workshop. Pre-session self-assessment surveys confirmed the ongoing relevance of identified learning needs. Postsession workshop evaluations informed continuous improvement, guiding facilitators in refining modules between cohorts using a Plan, Do, Study, Act approach (Fig. 2).

## Results:

Over 100 leaders (Quality Leaders, Interprofessional Education Specialists, Managers, Directors) across 12 different clinical areas attended the workshop series. Postsession survey results indicated participants valued the workshops in introducing and/or refreshing their understanding of their roles in enhancing the safety culture at SickKids. Employing a Plan, Do, Study, Act approach proved beneficial in ensuring the workshops’ effectiveness, with an increase likelihood between cohorts of participants recommending the program to a colleague.

## Conclusions:

A preliminary program evaluation suggests that to sustain consistent application of content, leaders will require ongoing support. A community of practice and dedicated time for refreshed training will be explored. Further evaluation of the program is underway, and with goals to expand the training beyond clinical operations, tailoring content will be required to reach a broader audience of clinical adjacent leaders.

